# Influence of Acrylamide Administration on the Neurochemical Characteristics of Enteric Nervous System (ENS) Neurons in the Porcine Duodenum

**DOI:** 10.3390/ijms21010015

**Published:** 2019-12-18

**Authors:** Katarzyna Palus, Jarosław Całka

**Affiliations:** Department of Clinical Physiology, Faculty of Veterinary Medicine, University of Warmia and Mazury in Olsztyn, Oczapowskiego Str. 13, 10-718 Olsztyn, Poland; calkaj@uwm.edu.pl

**Keywords:** acrylamide, duodenum, enteric nervous system, pig, neuroactive substances

## Abstract

The digestive tract, especially the small intestine, is one of the main routes of acrylamide absorption and is therefore highly exposed to the toxic effect of acrylamide contained in food. The aim of this experiment was to elucidate the effect of low (tolerable daily intake—TDI) and high (ten times higher than TDI) doses of acrylamide on the neurochemical phenotype of duodenal enteric nervous system (ENS) neurons using the pig as an animal model. The experiment was performed on 15 immature gilts of the Danish Landrace assigned to three experimental groups: control (C) group—pigs administered empty gelatine capsules, low dose (LD) group—pigs administered capsules with acrylamide at the TDI dose (0.5 μg/kg body weight (b.w.)/day), and the high dose (HD) group—pigs administered capsules with acrylamide at a ten times higher dose than the TDI (5 μg/kg b.w./day) with a morning feeding for 4 weeks. Administration of acrylamide, even in a low (TDI) dose, led to an increase in the percentage of enteric neurons immunoreactive to substance P (SP), calcitonin gene-related peptide (CGRP), galanin (GAL), neuronal nitric oxide synthase (nNOS), and vesicular acetylcholine transporter (VACHT) in the porcine duodenum. The severity of the changes clearly depended on the dose of acrylamide and the examined plexus. The obtained results suggest the participation of these neuroactive substances in acrylamide-inducted plasticity and the protection of ENS neurons, which may be an important line of defence from the harmful action of acrylamide.

## 1. Introduction

Acrylamide (ACM) is an organic chemical compound used in the manufacture of plastics, adhesives, masonry mortars, and cosmetics. ACM is also formed in the Maillard reaction during the thermal processing of food at high temperatures and its highest content was recorded in toasted bread, French fries, instant coffee, and chips [1]. Since the presence of ACM in food products has been confirmed, numerous toxicological studies have been conducted to investigate its effect on living organisms [2,3]. The digestive tract, especially the small intestine, is one of the main routes of acrylamide absorption and is therefore highly exposed to the toxic effect of acrylamide contained in food [4].

In the literature, there are many studies concerning the impact of acrylamide on living organisms. The genotoxic and carcinogenic effects of acrylamide have been described in many experimental models of acrylamide toxicity [5,6]. The toxic effects of acrylamide on the nervous tissue (damage of axons in the peripheral and central nervous system, inhibition of neurotransmitter release, and disturbed nerve conduction) have been also demonstrated in previous studies using rodent models [3,6]. The neurotoxicity of acrylamide has also been described in people exposed to acrylamide in factories as well as smokers [6]. Namely, in acute and subacute acrylamide intoxication, the symptoms of peripheral neuropathy have been observed. These are axonopathies, which initially include sensory and then motor fibers. The most common clinical symptoms include loss of sensation, numbness of hands and feet, muscle weakness, and decreased tendon reflexes [7]. Furthermore, acrylamide is a substance used in murine models of peripheral neuropathies [8]. However, despite numerous studies, there is a lack of data describing the impact of acrylamide on enteric nervous system (ENS) structures, especially in large animals.

Due to the great popularity of products containing significant amounts of acrylamide among consumers, the World Health Organization (WHO) recommends that food producers reduce the level of acrylamide in the finished products. However, until now, the maximum content of acrylamide in food is still not specified. This is particularly important because acrylamide passes through the placental barrier and into the milk [6]. It is important to mention that acrylamide doses used in toxicological studies with rodents are significantly higher than those consumed by humans. Daily exposure to acrylamide contained in food products in humans ranges between 0.3 to 0.8 µg/ kg of body weight [9]. In the current study, for the first time, the impact of acrylamide in tolerable daily intake (TDI) (0.5 µg/ kg of body weight (b.w.)/day) and ten times higher (5 µg/ kg of b.w./day) doses was examined, which reflects the actual intake of acrylamide in the human population. 

It is well known that the small intestine possesses two sources of innervation: neurons belonging to the ENS located in the wall of digestive tract and extrinsic sympathetic, parasympathetic, and sensory ganglia [10,11,12]. The anatomical structure and spatial distribution of ENS structures depends both on the part of the digestive tract and on the animal species. In pigs and other large animals, in the stomach, the ENS consist of two plexuses: the myenteric plexus (MP) and the submucous plexus (SP). In turn, in small and large intestines, two submucous plexuses—outer submucous plexus (OSP) and inner submucous plexus (ISP)—are present [13]. ENS neurons synthesize and secrete numerous neuroactive substances involved in the control of physiological functions in the digestive tract. Additionally, among the many neurotransmitters synthesized in ENS neurons, we can distinguish those involved in neuroprotective and defensive processes and those participating in the conduction of pain stimuli to the central nervous system (CNS) [10,11,12,13,14]. It should also be noted that the ENS is one of the first barriers of the organism against harmful substances present in food. The reaction of the ENS to pathological factors expressed as a change in the chemical coding of intramural neurons may be the first subclinical symptom of GI tract disorders. Previous reports revealed that gastrointestinal disturbances accompanying metabolic diseases, and naturally and experimentally induced inflammatory conditions and toxins, such as mycotoxins or bisphenol A, led to changes in the expression of neuroactive substances in ENS neurons within the intestines [15,16,17,18,19]. These changes are evidence of the high plasticity of the nervous system and may help recognize neuropeptides involved in protection of neurons from damage. Thus, the aim of this experiment was to elucidate the effect of low (TDI) and high (ten times higher than TDI) doses of acrylamide on the neurochemical phenotype of the duodenal ENS neurons using the pig as an animal model. 

## 2. Results

The administration of acrylamide even in low (TDI) dose led to changes in the percentage of enteric neurons immunoreactive to neuroactive substances studied in the porcine duodenum. The severity of the changes clearly depended on the dose of acrylamide and the examined plexus (Table 1). 

### 2.1. Myenteric Plexus (MP)

**Figure 1:**
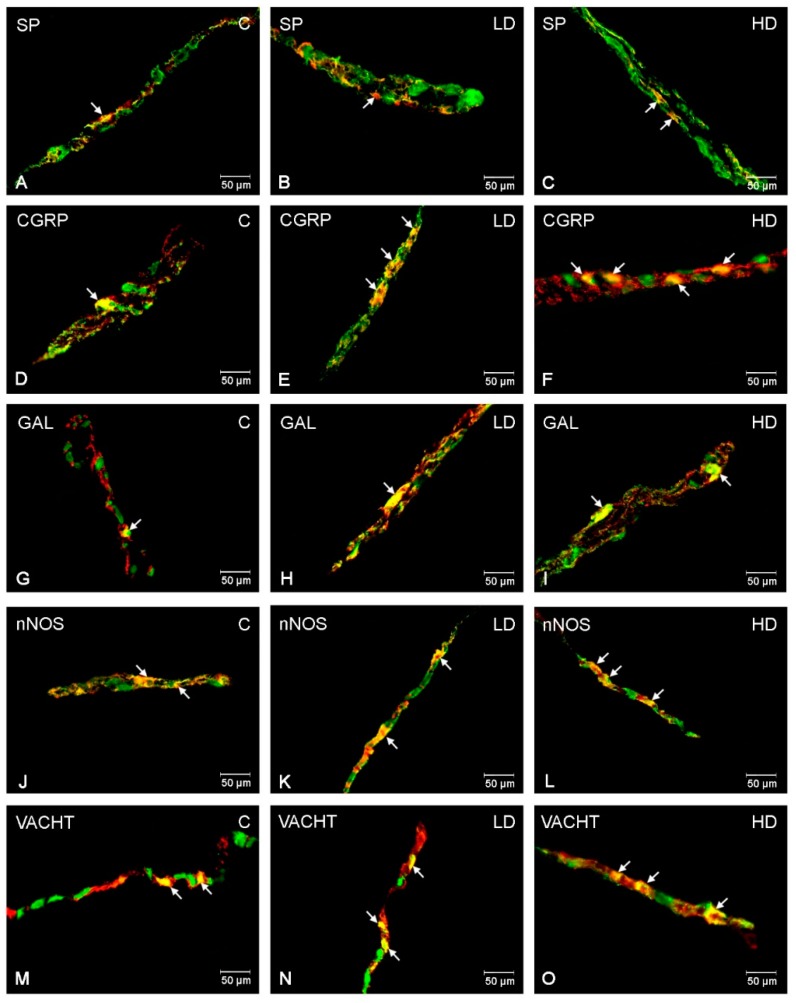
Immunofluorescence findings of ENS neurons in the myenteric plexuses. Representative images of duodenum myenteric neurons immunopositive to SP, CGRP, GAL, nNOS, and VACHT in physiological state (**A**,**D**,**G**,**J**,**M**), after low (**B**,**E**,**H**,**K**,**N**) and high (**C**,**F**,**I**,**L**,**O**) doses of acrylamide supplementation. (**A**–**C**)—myenteric neurons immunopositive to protein gene-product 9.5 (PGP9.5)—used as a panneuronal marker and SP; (**D**–**F**)—myenteric neurons immunopositive to PGP9.5 and CGRP, (**G**–**I**)—myenteric neurons immunopositive to PGP9.5 and GAL, (**J**–**L**)—myenteric neurons immunopositive to PGP9.5 and nNOS, and (**M**–**O**)—myenteric neurons immunopositive to PGP9.5 and to VACHT. All photographs have been made by overlapping of green and red fluorescent channels (green for PGP 9.5 and red for SP, CGRP, GAL, nNOS, and VACHT, respectively). Neurons immunopositive to particular substance studied are indicated with arrows.

In the control group, the most numerous populations of the ENS neurons were neuronal nitric oxide synthase (nNOS)-positive (29.34 ± 1.78%) (Figure 1C). A slightly lower number of myenteric neurons displayed vesicular acetylcholine transporter (VACHT)—(13.92 ± 0.91%) (Figure 1M) and calcitonin gene-related peptide (CGRP)—(12.38 ± 1.02%) (Figure 1D) immunoreactivity. In turn, galanin (GAL)-like immunoreactive (LI) (Figure 1G), as well as substance P (SP)-LI (Figure 1A), cell bodies constituted only a small percentage of all PGP 9.5-LI neurons (2.87 ± 0.41% and 0.69 ± 0.14%, respectively). Following acrylamide supplementation, an increase in the number of myenteric neurons immunoreactive to all neuroactive substances studied was observed (Table 1). The most significant changes were noted for CGRP, in which the increase was highly statistically significant in both experimental groups (to 21.75 ± 0.90% in the LD group and to 31.54 ± 0.70% in the HD group) (Figure 1E,F). Similarly, the percentage of GAL-LI neurons was significantly increased in the group receiving low (to 6.45 ± 0.70%) (Figure 1H) and high (to 24 ± 0.32%) (Figure 1I) doses of acrylamide. A slightly smaller increase was observed for VACHT (to 20.22 ± 0.46 and 24.89 ± 1.50%) (Figure 1N, O) and SP (to 1.05 ± 0.25 and 2.67 ± 0.44%) (Figure 1B,C), but the changes were also statistically significant in both groups. Only in the case of nNOS did a significant increase occur in animals receiving high doses of acrylamide (to 37.39 ± 0.98%) (Figure 1L).

### 2.2. Outer Submucous Plexus (OSP)

**Figure 2:**
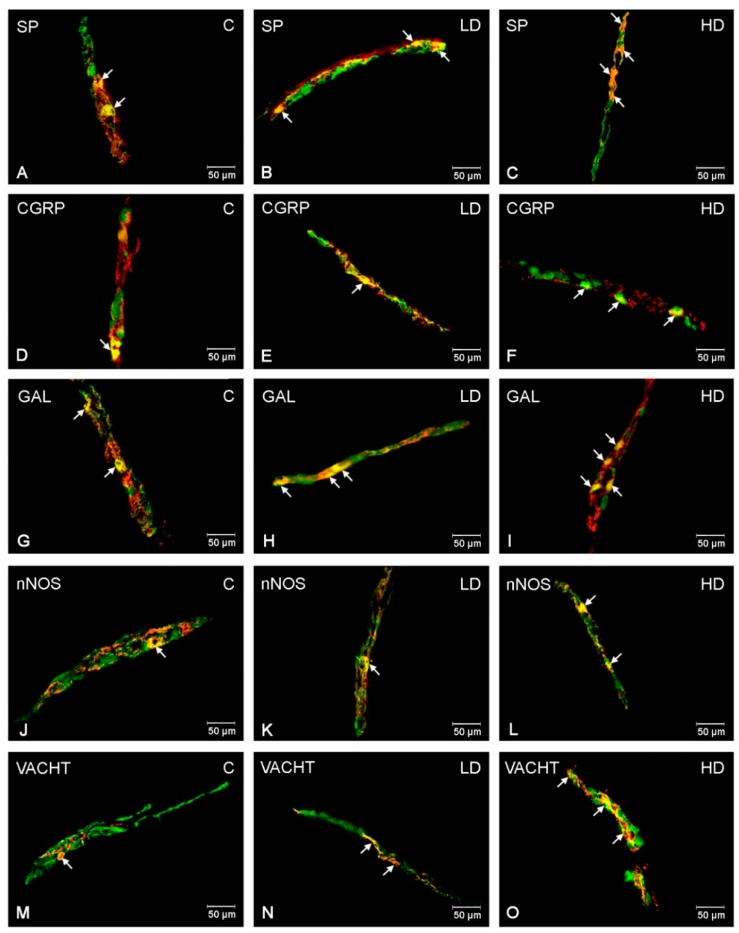
Immunofluorescence findings of the ENS neurons in the outer submucous plexuses. Representative images of duodenum other submucous neurons immunopositive to SP, CGRP, GAL, nNOS, and VACHT in physiological state (**A**,**D**,**G**,**J**,**M**), after low (**B**,**E**,**H**,**K**,**N**) and high (**C**,**F**,**I**,**L**,**O**) doses of acrylamide supplementation. (**A**–**C**)—outer submucous neurons immunopositive to protein gene-product 9.5 (PGP9.5)—used as a panneuronal marker and SP; (**D**–**F**)—outer submucous neurons immunopositive to PGP9.5 and CGRP, (**G**–**I**)—outer submucous neurons immunopositive to PGP9.5 and GAL, (**J**–**L**)—outer submucous neurons immunopositive to PGP9.5 and nNOS, and (**M**–**O**)—outer submucous neurons immunopositive to PGP9.5 and to VACHT. All photographs have been made by overlapping of green and red fluorescent channels (green for PGP 9.5 and red for SP, CGRP, GAL, nNOS, and VACHT, respectively). Neurons immunopositive to the particular substance studied are indicated with arrows.

Under physiological conditions, the highest number of OSP neurons in the porcine duodenum was GAL-positive (31.75 ± 1.41%) (Figure 2G), while SP- and VACHT-LI neurons constituted a slightly smaller group of neurons (21.47 ± 1.19% and 20.80 ± 1.00%, respectively) (Figure 2A,M). In turn, the number of CGRP-LI neurons was estimated at 14.62 ± 1.20% (Figure 2D). The least numerous groups among the examined neurons were nNOS-positive (2.41 ± 0.60%) (Figure 2J). The administration of acrylamide, in both low and high doses, led to a significant increase in the number of GAL-LI (39.10 ± 0.81% and to 49.78 ± 0.64%) (Figure 2H,I) and SP-LI (to 25.83 ± 1.28% and to 38.50 ± 1.23%) (Figure 2B,C) neurons. A slightly smaller, but also statistically significant, increase was noted in both experimental groups in the case of VACHT (to 26.88 ± 0.69% in LD group and to 33.60 ± 1.48% in the HD group) (Figure 2N,O) and nNOS (to 4.62 ± 0.28% in LD group and to 9.20 ± 0.46% in the HD group, respectively) (Figure 2K,L). For CGRP, the changes were significant only in the group receiving high doses of acrylamide (to 27.44 ± 0.99%) (Figure 2E,F).

### 2.3. Inner Submucous Plexus (ISP)

**Figure 3:**
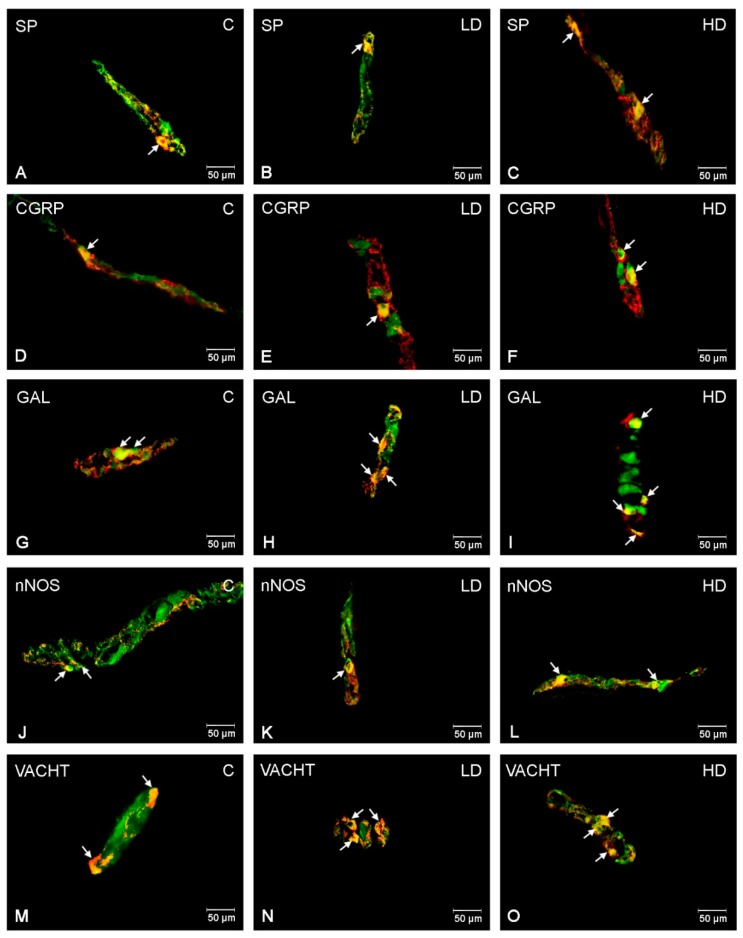
Immunofluorescence findings of the ENS neurons in the inner submucous plexuses. Representative images of duodenum inner submucous neurons immunopositive to SP, CGRP, GAL, nNOS, and VACHT in physiological state (**A**,**D**,**G**,**J**,**M**), after low (**B**,**E**,**H**,**K**,**N**) and high (**C**,**F**,**I**,**L**,**O**) doses of acrylamide supplementation. (**A**–**C**)—inner submucous neurons immunopositive to protein gene-product 9.5 (PGP9.5)—used as a panneuronal marker and SP; (**D**–**F**)—inner submucous neurons immunopositive to PGP9.5 and CGRP, (**G**–**I**)—inner submucous neurons immunopositive to PGP9.5 and GAL, (**J**–**L**)—inner submucous neurons immunopositive to PGP9.5 and nNOS, and (**M**–**O**)—inner submucous neurons immunopositive to PGP9.5 and to VACHT. All photographs have been made by overlapping of green and red fluorescent channels (green for PGP 9.5 and red for SP, CGRP, GAL, nNOS, and VACHT, respectively). Neurons immunopositive to a particular substance studied are indicated with arrows.

In the control group, the higher number of ISP neurons were GAL and VACHT-positive (42.74 ± 1.75% and 38.86 ± 1.53%, respectively) (Figure 3G,M). Next, SP-LI and CGRP-LI neurons represented a slightly smaller population of ISP neurons in the porcine duodenum (20.14 ± 0.69% and 11.57 ± 1.17%) (Figure 3A,D). In turn, only 1.53 ± 0.19% of all PGP 9.5 neurons were nNOS-positive (Figure 3J). After acrylamide supplementation, the most remarkable changes were observed for GAL and VACHT and a highly statistically important increase in the number of GAL-LI and VACHT-LI neurons was noted in both LD and HD groups (to 49.12 ± 1.15% and 54.45 ± 1.35% for GAL; to 52.81 ± 1.43% and 61.96 ± 1.06% for VACHT) (Figure 3H,I,N,O). An increase in the number of CGRP- and SP-LI cell bodies was also significant after low and high doses of acrylamide administration (to 22.59 ± 0.65% and 28.64 ± 0.77% in the case of CGRP; to 23.51 ± 1.18% and 26.77 ± 1.75% in the case of SP, respectively) (Figure 3B,C,E,F). However, for nNOS, only in the HD group was an increase statistically important (to 7.46 ± 0.65%) (Figure 3L). 

## 3. Discussion

The results of the present study have shown that SP, CGRP, GAL, nNOS, and VACHT were detected in all kinds of ENS plexuses (the MP, OSP, and ISP) in the porcine duodenum. This is in agreement with previous studies, where the presence of these substances was observed in enteric nervous structures, as well as extrinsic sources of the GI tract innervation, in numerous species, including humans [18,20,21,22,23,24]. This indicates that these substances are involved in the regulation of physiological processes in the porcine duodenum. This is consistent with the current knowledge regarding their physiological functions. In the digestive tract, substance P participates in the regulation of gastrointestinal motility, affects endothelial ion transport, and increases vascular permeability in inflammatory tissues [25]. SP is also involved in pain neurotransmission and the modulation of autonomic stimulus flow [26]. CGRP, as a marker of primary afferent neurons, is involved in pain conduction, but also plays an important role in the regulation of stomach contractility, secretion of hydrochloric acid, mucosal ion transport, and stimulates the release of somatostatin [27,28]. GAL is also thought to regulate numerous physiological actions in the mammalian digestive tract, including the secretion of digestive enzymes and hydrochloric acid secretion, regulation of peristalsis, and the release of other neurotransmitters [29]. Further, nNOS, a marker of nitrergic neurons, is an inhibitory neurotransmitter responsible for the slowing of intestinal motility, suppression of the release of other neurotransmitters, and the control of blood flow in intestinal blood vessels [30]. In turn, VACHT, as a cholinergic component, stimulates smooth muscle contractility and the secretion of digestive enzymes [31]. 

This study also provided an evaluation of changes in the number of enteric neuronal populations in the intramural plexuses of the pig duodenum following acrylamide administration. Alterations in the number of ENS neurons displaying immunoreactivity to all substances under investigation were observed in each kind of enteric plexus studied. This is congruent with the phenomenon of neural plasticity, described by many authors as an adaptation in ENS neurons to disturbances of homeostasis and pathological factors manifested by the change of neurochemical features of neurons, overexpression of certain genes, or a reduction in the expression of others [32]. The plasticity of ENS neurons has been confirmed in studies demonstrating the effect of pathological gut conditions, intoxications, and other experimental treatments in numerous animal models and humans [13,15,18,19,21,25,33].

However, the toxicity of acrylamide was confirmed in previous research using experimental animals. In rodents, the consumption of acrylamide potentially led to cancers of various organs such as testes, ovaries, breast, GI tract, kidney, and lung, as well as reproductive disorders and peripheral neuropathy [3,6]. To date, a carcinogenic effect has not been confirmed in humans [34]. We can speculate that these differences are related to the doses of acrylamide used in previous experiments with rodents, which significantly exceeded human consumption of this toxin. Nonetheless, there is credible scientific evidence of the neurotoxic effects of acrylamide on the human body. It has been shown that acrylamide can react with cysteine residues in the presynaptic membrane protein and reduces the release of neurotransmitters, which leads to neuronal degeneration [6]. Inhibition of creatine kinase activity in the brain and sciatic nerve has also been reported, resulting in a deficiency of ATP in cells, leading to their death [7]. Additionally, inhibition of fast axonal transport is also one of the main mechanisms of acrylamide neurotoxicity [35]. It was also shown that fetuses of pregnant mothers receiving acrylamide showed degenerative changes in the brain, reducing the level of trophic factors and hemorrhagic damage [36]. Furthermore, Lo Pachin et al. [37] reported that acrylamide affects nerve terminals, leading to autonomic, sensory, and motor disturbances. Purkinje cell damage is also an important symptom of acrylamide intoxication. Other authors pointed out that acrylamide intoxication is involved in dysfunction of the dopaminergic system [38]. Interestingly, Lee et al. [39] demonstrated that acrylamide led to developmental neurotoxicity by a delay of maturation of primary cultured neurons. 

Previous findings also demonstrated the involvement of oxidative stress in the toxicity of acrylamide [40]. In vitro studies have shown an increase in reactive oxygen species (ROS) and a reduction of cellular concentration of the reduced form of glutathione (GSH) in isolated human monocytes and HepG2 cells [41]. The activity of superoxide dismutase (SOD) was elevated in the liver, testes, kidney, and lung of rats during acrylamide administration and in human erythrocytes treated with acrylamide in vitro [42]. It has been shown that acrylamide, by binding to GSH stores, led to a change in the redox status of the cell and, consequently, induced apoptosis [43]. Acrylamide-induced oxidative stress may also help to mediate the activation of glial cells and the release of proinflammatory cytokines, consequently leading to neuronal damage [44]. This hypothesis is supported by numerous studies using natural and synthetic antioxidants (including vitamin C, vitamin E, green tea, and others) in the protection of neurons by acrylamide neurotoxicity [45,46,47]. Oxidative stress and a deficiency of antioxidants may also play an important role in GI tract damage caused by acrylamide. 

Knowledge concerning the effect of acrylamide on the gut, especially on the ENS, is scarce. Based on data from the central and peripheral nervous systems, it is speculated that changes observed in the ENS are a result of its neurotoxic effects. It is also in line with previous study of Tomaszewska et al. [48] describing the negative effect of acrylamide administration on small intestine histomorphometry, including ENS structures. This hypothesis is supported by the fact that most of the neuroactive substances used in the present experiment have neuroprotective properties. Firstly, the neurotrophic and growth-promoting role of GAL was confirmed in numerous studies concerning neuronal degeneration or injury in different types of neurons [29]. Secondly, over-expression of nNOS in myenteric neurons has been described in many pathological processes in the gut, which suggests its participation in neuroprotective and recovery processes [49]. Further, an elevated number of VACHT-LI enteric neurons were also found in numerous GI tract disorders [18,50]. Unquestionably, ACh displays neuroprotective activity in different kinds of neuronal structures [51]. In turn, SP and CGRP are engaged in the conduction of pain accompanying neuronal damage or injury [26,27]. However, further toxicological research is needed to elucidate the mechanisms of the neurotoxic impact of acrylamide on ENS neurons. 

It is also suspected that the observed changes are associated with inflammatory conditions accompanying acrylamide administration. It has been shown that high doses of acrylamide contained in potato chips led to activation of inflammatory responses in the CNS of albino mice and release of pro-inflammatory cytokines, including interleukin 1β (IL-1β), interleukin 6 (IL-6), interleukin 18 (IL-18), and the inducible form of nitric oxide synthase (iNOS) [52]. A similar observation was noted in humans, in which the consumption of potato chips resulted in an elevated level of inflammatory indicators (IL-6, C-reactive protein (CRP)) as well as an increase in cholesterol (LDH) content in the blood [53]. In a previous report, the authors also confirmed that acrylamide intoxication led to a local inflammatory state in the porcine ileum wall and the release of pro-inflammatory cytokines in the ileal Payer patches (IL-6, IL-1β, and tumour necrosis factor-α (TNF-α)) [54]. The obtained results are in line with previous reports in which upregulated expressions of neuroactive substances used in the present study in natural and experimentally inducted inflammatory states were also reported. SP is one of the major factors involved in the regulation of inflammatory conditions in the GI tract via activation of NK1 receptors [55]. An increased population of SP-LI intramural neurons was observed following childhood chronic gastritis and duodenitis, gastric ulcers, and *Helicobacter pylori* infection [56,57,58]. Co-operation between SP and the immune cells has also been reported. SP modulates the inflammatory response and leads to increased synthesis of interleukin-1 (IL-1), IL-6, IL-8, and TNF-α [59]. Similarly, CGRP is involved in the regulation of cytokines secretion and leads to a decrease in the level of TNF-α and IL-1β [60]. Upregulated expression of CGRP was noted during numerous pathological states in the GI tract, including peptic ulcers, chemically induced inflammation of porcine descending colon, and ulcerative colitis [61,62,63]. Additionally, as a sensory neuropeptide, it is unquestionably involved in the conduction of pain accompanying the inflammation [28]. Further, numerous reports confirm the involvement of GAL in the control of inflammatory processes in the digestive tract [29,64]. An increase in the population of GAL-LI neurons was observed in structures of the ENS, as well as extrinsic sources of innervation of the GI tract, following colitis, gastric ulcers, hyperacidity of the stomach, and enteric Salmonella infection [24,64,65,66]. Moreover, GAL triggers an immunological system response and modulates the synthesis of pro-inflammatory mediators, including TNF-α, IL-1α, and IL-8 [67]. In addition, in the case of VACHT, many papers describing elevated expression of this substance during gut inflammation have recently been published [49,68,69]. Leite et al. [68] demonstrated that VACHT is involved in inflammatory response induced by lipopolysaccharide (LPS). The anti-inflammatory action of ACh was also observed in human blood macrophages during LPS administration expressed as an elevated level of TNF-α, IL-6, IL-18 [69]. In turn, nNOS may play both anti- and pro-inflammatory roles. An increased population of NOS-LI neurons in the ENS was described in inflammatory bowel disease (IBD), Crohn’s disease, and bisphenol A intoxication [18,70,71]. However, a decrease in nNOS immunoreactivity was observed in Crohn’s disease, inflammatory processes, and diabetes [19,72,73]. The nNOS function probably depends on both the localization in the GI tract and type of inflammation. The current results supported by previous reports indicate that SP, CGRP, GAL, nNOS, and VACHT are important factors involved in the control of inflammatory states in the gut and may be engaged to protect ENS neurons against acrylamide-induced inflammatory conditions. 

It should also be noted that even a low dose of acrylamide triggered a significant response by ENS neurons. The obtained results raise the question if the consumer-acceptable doses of acrylamide are safe for human health, especially for children. It is therefore necessary to analyze the doses of acrylamide and to strive for the maximum reduction of acrylamide in food products, especially since the increase in the pace of life and consumption of articles containing acrylamide is growing. Furthermore, due to difficulties in assessing the neurotoxicity of acrylamide in the human body, it is very important to use appropriate animal models. The pig is an omnivorous animal whose anatomical and histological structure is highly comparable with the human body [74]. In addition, physiological and pathological processes, as well as microflora, are more like those found in rodents [75]. Earlier reports also confirm that the pig is a great model for biomedical research concerning gastrointestinal disorders [76]. This makes the pig an extremely useful model in understanding the effects of acrylamide on the ENS. 

## 4. Materials and Methods

### 4.1. Animals and Experimental Procedures

The experiment was performed on 15 immature gilts of the Danish Landrace (8 weeks old, about 20 kg of body weight (b.w.)), as described previously by Palus et al. [77]. Pigs from all groups were kept under standard laboratory conditions, fed with commercial feed for pigs of this age group and had free access to water. After a one-week adaptive period, the pigs were divided into three experimental groups: control (C group, *n* = 5)—pigs administrated empty gelatine capsules, a low dose group (LD group, *n* = 5)—pigs administrated capsules with acrylamide (>99%; Sigma-Aldrich, Saint Louis, MI, USA) at the tolerable daily intake (TDI) (0.5 μg/kg b.w./day), and the high dose group (HD group, *n* = 5)—pigs administrated capsules with acrylamide at a ten times higher dose than the TDI (5 μg/kg b.w./day). All experimental procedures were approved by the Local Ethical Committee for Experiments on Animals in Olsztyn (Approval No.: 28 February 2017). To ensure the appropriate dose of acrylamide, all pigs were weighed once a week. Capsules were administrated with the morning meal for 28 days. After a supplementation period, all pigs were pre-medicated with azaperone (Stresnil, Jansen Pharmaceutica N.V., Belgium, 4 mg/kg of body weight, intramuscularly (i.m.)) and then euthanized with a lethal dose of sodium pentobarbital (Morbital, Biowet Puławy, Puławy, Poland; 0.6 mL/kg of body weight, intravenously (i.v.)). After the cessation of vital functions, duodenum fragments (about 3 cm in length) located 10 cm caudal to the gastric pylorus were collected from each animal. All fragments were then fixed in a solution of a 4% buffered solution of paraformaldehyde (pH = 7.4) for 1 h, rinsed three times in 0.1 M phosphate buffer (pH 7.4, every 24 h), put into 18% buffered solution of sucrose (pH = 7.4), and stored for 14 days. 

### 4.2. Double-Labelling Immunofluorescence 

Microscopic sections (14 µ thick) of duodenum samples were double immunostained using primary antisera and appropriate secondary antibodies (Table 2). Sections were dried for 45 min (at 20 °C) and rinsed three times in 0.1 M phosphate-buffered saline (PBS, pH = 7.4, 10 min). Next, they were incubated (1 h) with a blocking solution (containing 10% horse serum and 0.1% bovine serum albumin in 0.1 M PBS, 1% Triton X-100, 0.05% Thimerosal, and 0.01% sodium azide), rinsed in PBS (3 times × 10 min), and incubated overnight with primary antisera against pan-neuronal marker PGP 9.5 and substance P (SP), calcitonin gene-related peptide (CGRP), galanin (GAL), neuronal nitric oxide synthase (nNOS), vesicular acetylcholine transporter (VACHT) (Table 2). The next day, sections were rinsed in PBS (3 times × 10 min) and incubated with corresponding secondary antibodies (Table 2) (1 h, at room temperature). After another rinsing in PBS (3 times × 10 min), sections were cover-slipped in carbonate-buffered glycerol (pH 8.6). A negative control, including the pre-absorption, the omission, and the replacement tests, was also performed, which completely eliminated nonspecific staining.

### 4.3. Counting and Statistics

The number of neurons immunopositive to particular neuroactive substances studied was evaluated by counting neurons immunoreactive to these substances and numbers of PGP 9.5-LI neurons in all types of duodenum plexuses. For all substances studied, a minimum of 500 PGP 9.5-LI neurons with clearly visible nuclei was counted. The number of SP-, CGRP-, GAL-, VACHT-, and nNOS-positive neurons was estimated as a percentage of PGP 9.5 neurons (corresponding to the entire studied population of neurons). Additionally, sections selected for the present study were separated by at least 200 µm from each other to avoid double-counting of the same neuroactive substance. Results of present investigations were pooled and presented as a mean ± standard error of mean (SEM). The statistically important differences were estimated using Statistica 12 (Stat Soft Inc., Tulsa, OK, USA). One-way analysis of variance (ANOVA) with Dunnett’s test was used (* *p* < 0.05, ** *p* < 0.01, *** *p* < 0.001).

## 5. Conclusions

Acrylamide administration led to significant changes in the expression of all neuroactive substances studied in ENS neurons of the porcine duodenum. An increase in the number of SP-, CGRP-, GAL-, nNOS-, and VACHT-LI neurons was noted in all kinds of intramural neurons. It is suspected that the observed changes resulted from the neurotoxic effect of acrylamide on the ENS. They may also be related with the pro-inflammatory properties of acrylamide. In view of the previous reports supported by the current results, it may be expected that the participation of these neuroactive substances in acrylamide-inducted plasticity and the protection of ENS neurons may be an important line of defence from the harmful action of acrylamide. However, more detailed toxicological and clinical studies are necessary to better understand this issue.

## Figures and Tables

**Table 1:** Percentage of neurons immunoreactive to individual neuroactive substance studied in the porcine duodenum in the control group (C group) and after low (LD group) and high (HD group) doses of acrylamide supplementation.

	MP			OSP			ISP		
Experimental Group	C Group	LD Group	HD Group	C Group	LD Group	HD Group	C Group	LD Group	HD Group
**SP**	0.69 ± 0.14	1.05 ± 0.25 (**)	2.67 ± 0.44 (**)	21.47 ± 1.19	25.83 ± 1.28 (***)	38.50 ± 1.23 (***)	11.57± 1.17	23.51± 1.18 (**)	26.77± 1.75 (***)
**CGRP**	12.38 ± 1.02	21.75 ± 0.90 (***)	31.54 ±0.70 (***)	14.62 ± 1.20	16.15 ± 0.68	27.44 ± 0.99 (***)	20.14 ± 0.69	22.59 ± 0.65 (*)	28.64 ± 0.77 (***)
**GAL**	2.87 ± 0.41	6.45 ± 0.70 (***)	10.24 ± 0.32 (***)	31.75 ± 1.41	39.10 ± 0.81 (***)	49.78 ± 0.64 (***)	38.86 ± 1.53	49.12 ± 1.15 (***)	54.45± 1.35 (***)
**nNOS**	29.34 ± 1.78	31.55 ± 1.12	37.39 ± 0.98 (**)	2.41 ± 0.60	4.62 ± 0.28 (*)	9.20 ± 0.46 (***)	1.53 ± 0.19	2.84 ± 0.26	7.46 ± 0.65 (***)
**VACHT**	13.92 ± 0.91	20.22 ± 0.46 (**)	24.89 ± 1.50 (***)	20.80 ± 1.00	26.88 ± 0.69 (**)	33.60 ± 1.48 (***)	42.74 ± 1.75	52.81 ± 1.43 (**)	61.96 ± 1.06 (***)

MP—myenteric plexus, OSP—outer submucous plexus, ISP—inner submucous plexus, SP—substance P, CGRP—calcitonin gene-related peptide, GAL—galanin, nNOS—neuronal nitric oxide synthase, VACHT—vesicular acetylcholine transporter. * *p* < 0.05, ** *p* < 0.01, *** *p* < 0.001.

**Table 2:** Primary and secondary antibodies used in immunofluorescence technique.

Antigen	Host Species	Cat No.	Dilution	Supplier
**Primary Antibodies**				
PGP 9.5	Mouse	7863-2004	1:1000	Bio-Rad, Hercules, CA, USA
SP	Rat	8450-0505	1:150	AbD Serotec, Raleigh, NC, USA
CGRP	Rabbit	MAB317	1:4000	Millipore, Burlington, MA, USA
GAL	Rabbit	RIN7153	1:3000	Peninsula, San Carlos, CA, USA,
nNOS	Rabbit	AB5380	1:2000	Sigma-Aldrich, Saint Louis, MO, USA
VACHT	Rabbit	H-V007	1:2000	Phoenix Pharmaceuticals, Burlingame, CA, USA
**Secondary Antibodies**				
Alexa Fluor 488 donkey anti-mouse IgG		A21202	1:1000	Thermo Fisher Scientific, Waltham, MA, USA
Alexa Fluor 546 goat anti-rabbit IgG		A11010	1:1000	Thermo Fisher Scientific, Waltham, MA, USA
Alexa Fluor 546 goat anti-rat IgG		A11081	1:1000	Thermo Fisher Scientific, Waltham, MA, USA
